# Size Dependency of Post-Disturbance Recovery of Multi-Stemmed Resprouting Trees

**DOI:** 10.1371/journal.pone.0105600

**Published:** 2014-08-21

**Authors:** Jennifer L. Schafer, Michael G. Just

**Affiliations:** Department of Plant and Microbial Biology, North Carolina State University, Raleigh, North Carolina, United States of America; Ecole Pratique des Hautes Etudes, France

## Abstract

In frequently burned ecosystems, many plants persist by repeated resprouting from basal or belowground buds. This strategy requires that plants reach a balance between biomass loss and recovery, which depends on the shape of the relationship between pre- and post-fire size. Previous analyses of this relationship, however, have focused on the size of the largest stem, which ignores the importance of the multi-stem growth habit that is common in pyrogenic ecosystems. We hypothesized that the presence of multiple stems causes a substantial shift in the relationship between pre- and post-fire size and in the relationship between pre-fire size and size recovery. We measured the height and basal diameter, then calculated volume and biomass, of all stems of six tree species before and nine months after complete removal of aboveground biomass via coppicing. The number of resprouts was correlated with the original number of stems for four species. For all species, the relationship between pre-coppicing and resprout size fit a positive curvilinear function, and the shape of this curve did not differ for maximum and total stem size. Smaller individuals recovered a larger proportion of their pre-coppicing size than larger individuals, but the shape of the size recovery curves were the same regardless of whether the analysis was performed with all stems or only the largest stem. Our results indicate that measuring only the largest stem of multi-stemmed individuals is sufficient to assess the ability of individuals to recover after complete loss of aboveground biomass and persist under frequent burning.

## Introduction

Resprouting provides resilience to fire and allows plants to persist in pyrogenic ecosystems. When aboveground stems are killed by fire (i.e., topkilled), species that are able to resprout generate new biomass from plant parts that survive fire [1], [2] such as basal buds, lignotubers, rhizomes, or the root collar [3], [4]. Resprouting ability and resprout biomass [5]–[7] are influenced by the size of the belowground bud bank [8], the pool of belowground resources (e.g., carbohydrates and nutrients [9]–[14]), and pre-fire plant size [15], [16].

**Figure 1:**
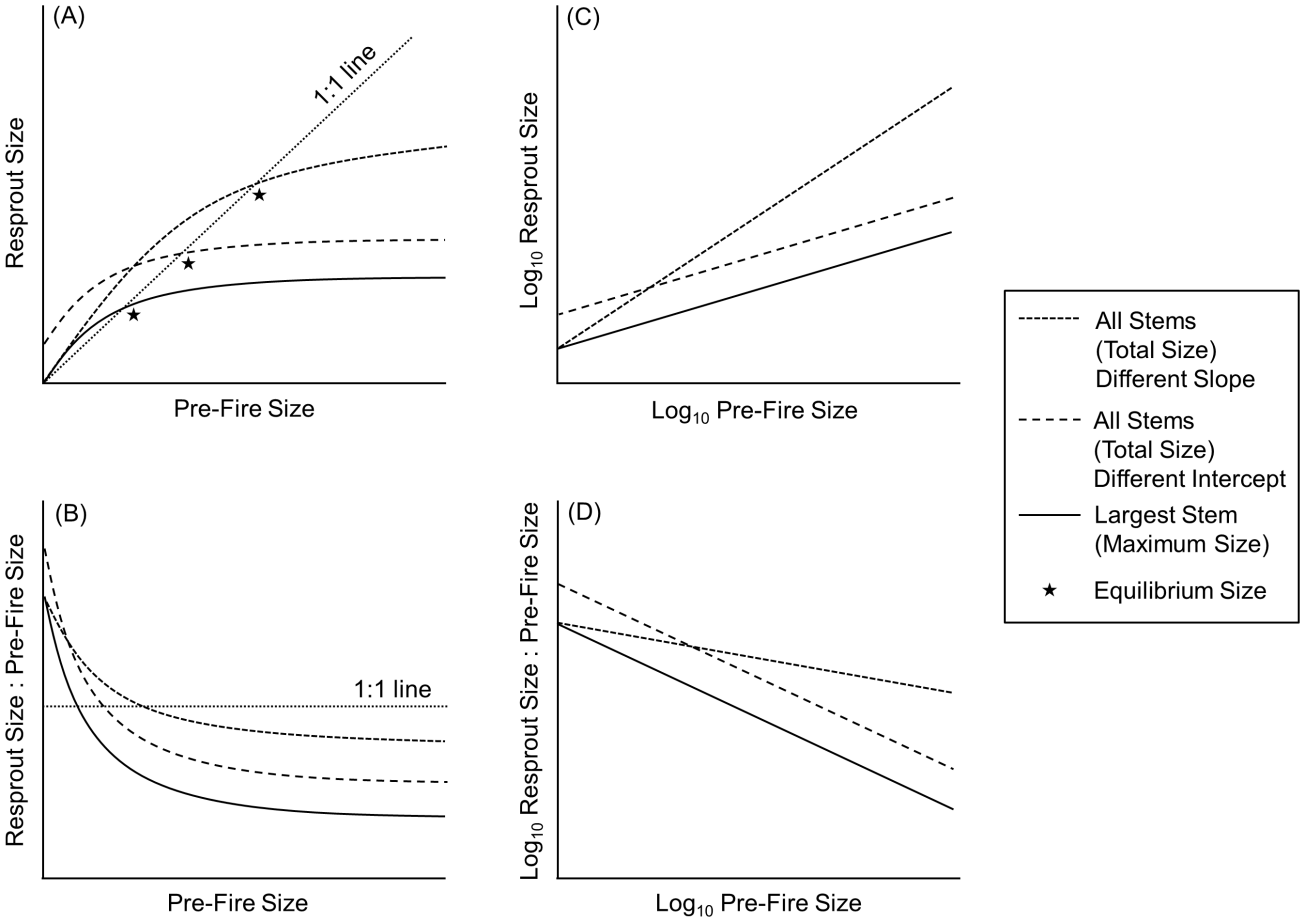
Hypothesized shifts in resprout and recovery curves resulting from inclusion of all stems. (A) Differences in resprout curves that could arise from inclusion of all stems of multi-stemmed trees. Stars indicate the equilibrium size that develops over multiple fire cycles that corresponds to the point at which biomass loss is equal to biomass recovery (i.e., intersects with the 1∶1 line; following [18]). (B) Recovery curves that correspond with resprout curves. (C) Illustration of the transformation of resprout curves to a logarithmic scale. (D) Illustration of the transformation of recovery curves to a logarithmic scale. We assessed shifts in resprout and recovery curves by testing for differences in the slopes and y-intercepts of the log_10_-transformed relationships between maximum and total size and size recovery.

In frequently burned ecosystems, resprouting species are subjected to repeated cycles of topkill and resprouting [17], so persistence depends on the ability of plants to recover their pre-fire size to maintain a balance between biomass loss and recovery [13], [18]. Resprout height and diameter are positively correlated with pre-fire stem height and diameter [16], [19], [20], with the relationship between pre- and post-fire size fitting a curvilinear scaling function [18]. This “resprout curve” illustrates the balance between biomass loss and recovery and determines the equilibrium size (i.e., where pre-fire and post-fire size are equal) upon which plants will converge over multiple fire cycles ([18]; Figure 1A). Although resprout size is correlated with pre-fire size [16], [19], [20], large plants often recover their pre-fire size more slowly than small plants [18], [21]. This “recovery curve” is a negative curvilinear relationship between pre-fire size and the ratio of post- to pre-fire size (Figure 1B).

Studies on the relationship between pre- and post-fire size and the size dependency of post-fire recovery, however, often focus only on the largest pre-fire stem and largest resprout [16], [18], [19] even though many resprouting species are multi-stemmed before and/or after fire (e.g., [22]–[24]). In fact, the number of resprouts is correlated with the number of stems pre-fire [20], [25], [26]. Allocation of biomass to multiple stems, rather than one stem, may be beneficial due to limitations on maximum stem height and growth rates [27]–[29] and the improvement in competitive success conferred by a large crown volume [30]. If the curvilinear nature of resprout and recovery curves is a consequence of limitations on maximum stem growth rates [28], [31], then this limitation could be overcome by producing multiple stems.

Accounting for all stems of multi-stemmed resprouting species, therefore, may cause an upward shift in resprout (Figure 1A) and recovery (Figure 1B) curves. An upward shift in the resprout curve would indicate that individual plants are able to maintain a greater biomass (i.e., a greater equilibrium plant size) with frequent burning. Consequently, larger individuals would be able to recover their pre-fire size. In this case, production of multiple stems could increase the ability of plants to escape a suppressed state of repeated topkill and resprouting [17], [32], [33] during a longer fire free interval. Alternatively, accounting for all stems could lead to a change from a curvilinear to linear relationship between pre- and post-fire size and size recovery, indicating that curvilinearity is not a fundamental property of resprouting. Regardless, understanding the impact of multiple stems on resprout and recovery curves is important because the ability of individual plants to recover biomass lost during fire allows for persistence with repeated burning [13].

We assessed resprouting success and the size dependency of volume and biomass recovery after complete loss of aboveground biomass. Specifically, we coppiced aboveground stems – as has been done in other studies to simulate fire-induced topkill [6], [14], [34], [35] – of six tree species that occur in the pyrogenic longleaf pine savannas and adjacent stream-head pocosins of the southeastern United States [36], [37]. To test the hypothesis that accounting for all stems of multi-stemmed resprouting species causes a shift in resprout and recovery curves, we measured all stems pre-coppicing and all resprouts. We assessed possible shifts in resprout and recovery curves by testing for differences in the slopes and y-intercepts of the log-transformed relationships between pre-coppicing and resprout size (i.e., volume and biomass) of the largest stem (maximum size) and all stems (total size; Figures 1C and 1D).

## Materials and Methods

### Ethics Statement

We obtained approval for data collection from the Endangered Species Branch at Fort Bragg Military Installation. Data was collected on publicly owned land. No protected species were used in this study.

### Study site and species

We conducted our study at Fort Bragg, which is located in the Sandhills region of North Carolina (35° 07′N, 79° 10′W). Longleaf pine (*Pinus palustris* Mill.) savanna (e.g., upland pine/scrub oak sandhill *sensu* [36]) is the most widespread vegetation type on the installation. Pine needles and wiregrass (*Aristida stricta* Michx.) accumulate quickly and facilitate frequent fire; the average historical fire return interval is approximately 2 years [38]. For the lowland stream-head pocosins (i.e., wetlands) embedded within the savanna matrix [37], higher moisture content and differences in species composition contribute to a longer fire return interval of 7–50 years [39]. Soil moisture increases along the gradient from upland savanna to lowland pocosin, and soils are classified as entisols, inceptosols, or ultisols [40]. Mean annual precipitation is 1275 mm, and summer is the wettest season [37]. Fort Bragg is divided into discrete landscape units locally referred to as burn blocks, within which prescribed fire is applied approximately every 3 years [41]. Prescribed fires at Fort Bragg are conducted during the dormant season (December–March) and the growing season (April–July) [42]. Lightning ignited fires in the southeastern US typically occur during the spring and summer (April–September) [43], [44].

We selected six focal species that differ in their distribution along the savanna-to-pocosin gradient: *Quercus laevis* Walter (turkey oak), *Diospyros virginiana* L. (persimmon), *Liquidambar styraciflua* L. (sweetgum), *Liriodendron tulipifera* L. (tulip poplar), *Persea palustris* (Raf.) Sarg. (swamp bay), and *Acer rubrum* L. (red maple; Table 1; nomenclature follows The PLANTS Database (US Department of Agriculture, Natural Resources Conservation Service; http://plants.usda.gov/java/). *Quercus laevis* is a savanna species, and *D. virginiana* occurs in the savanna and the ecotone between savanna and pocosin. *Liquidambar styraciflua* is most common in the ecotone, and *L. tulipifera*, *P. palustris*, and *A. rubrum* are restricted to the pocosin and ecotone. All study species typically resprout from basal or belowground buds after topkill via fire or other damage.

**Table 1:** General characteristics of individuals included in the study.

Species^a^	# Individuals Coppiced^b^	# Individuals Resprouted	# of Stems Pre-Coppicing	# of Resprouts	Maximum Stem Height (cm)^c^	Maximum Basal Diameter (mm)^c^
*Quercus laevis*	29	26	1–6	0–8	60–330	6.99–61.18
*Diospyros virginiana*	26	26	1–2	1–5	58–285	7.74–40.01
*Liquidambar styraciflua*	32	31	1–5	0–17	12–399	2.38–41.20
*Liriodendron tulipifera*	29	27	1–8	0–37	37–610	5.12–56.92
*Persea palustris*	31	31	1–4	1–7	53–289	5.46–34.33
*Acer rubrum*	30	28	1–6	0–9	38–382	4.27–30.13

a Species are listed in order of their position along the savanna-to-pocosin gradient.

b Does not include individuals burned in the June 2013 wildfire.

c For stems pre-coppicing.

### Field measurements and calculations

In October 2012, we selected 30–32 individuals of each species that spanned a range of maximum stem heights and diameters (Table 1); even the largest individuals were considered saplings. Individuals were found throughout their distribution along the savanna-to-pocosin gradient in multiple burn blocks (n = 6) that were burned 3–4 years previously. We measured the height and basal diameter (within 2 cm of ground level) of all stems of each individual and then, to simulate topkill, coppiced all stems at ∼2 cm above ground level. Stems were coppiced in October, at the beginning of the dormant season and after the typical wildfire season [43], [44]. Coppicing is commonly used as a surrogate for disturbance-induced topkill (e.g., [34], [35]), and resprouting success is similar between burned and coppiced individuals [6], [15], [45]. A wildfire in June 2013 burned a small section of one of our study sites, which reduced our sample size of all species except *L*. *styraciflua* (Table 1). In July 2013, 9 months after coppicing and near the end of the growing season, we measured the height and basal diameter of all resprouts of each individual.

For individuals that resprouted, we calculated the conical volume of each stem from measurements of stem height and diameter. We determined the maximum stem volume (i.e., volume of the largest stem) and total stem volume (i.e., the sum of volumes of all stems) of each individual pre-coppicing and after resprouting. For three species – *Q. laevis*, *D. virginiana*, and *L. styraciflua* – we used allometric equations from Robertson and Ostertag [46] to calculate maximum and total stem biomass of each individual pre-coppicing and after resprouting. Except for large pre-coppiced *Q. laevis* and small *L. styraciflua* resprouts, the majority of our stems were within the range of diameters used to develop the allometric equations [46].

### Statistical analyses

**Figure 2:**
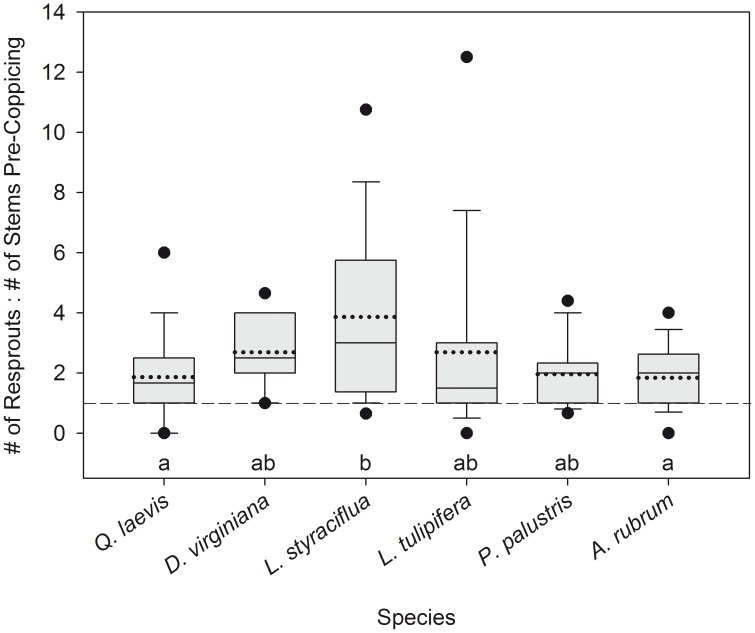
Ratio of resprout stem number to pre-coppicing stem number. In the boxplots, the solid and dotted bars represent the median and mean, respectively; the lower and upper bars represent the 25^th^ and 75^th^ percentiles, respectively. The lower and upper “whiskers” show the largest and smallest values that are not outliers and the lower and upper dots show the 5^th^ and 95^th^ percentiles. Kruskal-Wallis Χ^2^ = 20.03, *P* = 0.001; different letters indicate significant differences among species. The dashed line indicates where the number of resprouts equals the number of stems pre-coppicing.

For each species, we analyzed the relationship between pre-coppicing stem number and the number of resprouts using Kendall's tau. To analyze differences among species in resprouting success (i.e., production of resprouts), we calculated the ratio of resprouts to pre-coppicing stems for all individuals and used a Kruskal-Wallis test, with post-hoc pair-wise significance tests adjusted for multiple comparisons. To determine if there was a significant curvilinear relationship between total resprout size and total pre-coppicing size, we used the curve estimation function in SPSS version 19.0 (IBM Corporation, Armonk, NY, USA). Specifically, we fit power functions to the relationships between total pre-coppicing stem volume and total resprout stem volume (for all species) and total pre-coppicing stem biomass and total resprout biomass (for *Q. laevis*, *D. virginiana*, and *L. styraciflua*). We used a regression model with log_10_ resprout size (i.e., volume or biomass) as the dependent variable and log_10_ pre-coppicing size (i.e., volume or biomass), data type (i.e., maximum or total size, coded as 1 and 0, respectively), and an interaction term (log_10_ pre-coppicing size * data type) as independent variables entered into the model to determine if there was a significant difference between the slopes and y-intercepts of the relationships between: (1) pre-coppicing and resprout maximum and total stem size (Figure 1C) and (2) pre-coppicing size and recovery of maximum and total stem size (Figure 1D) for each species.

## Results

For all species, at least 90% of individuals resprouted after coppicing (Table 1). The number of resprouts was positively correlated with the number of coppiced stems for *Q. laevis* (τ = 0.339, *P* = 0.025), *L. styraciflua* (τ = 0.296, *P* = 0.042), *P. palustris* (τ = 0.296, *P* = 0.058), and *A. rubrum* (τ = 0.491, *P* = 0.001). The number of resprouts of *D. virginiana* (τ = 0.251, *P* = 0.239) and *L. tulipifera* (τ = 0.224, *P* = 0.139) was not correlated with the number of coppiced stems. The number of resprouts of all species tended to be equal to or greater than the number of coppiced stems (Figure 2); 73% of individuals that resprouted had more resprouts than coppiced stems.

**Figure 3:**
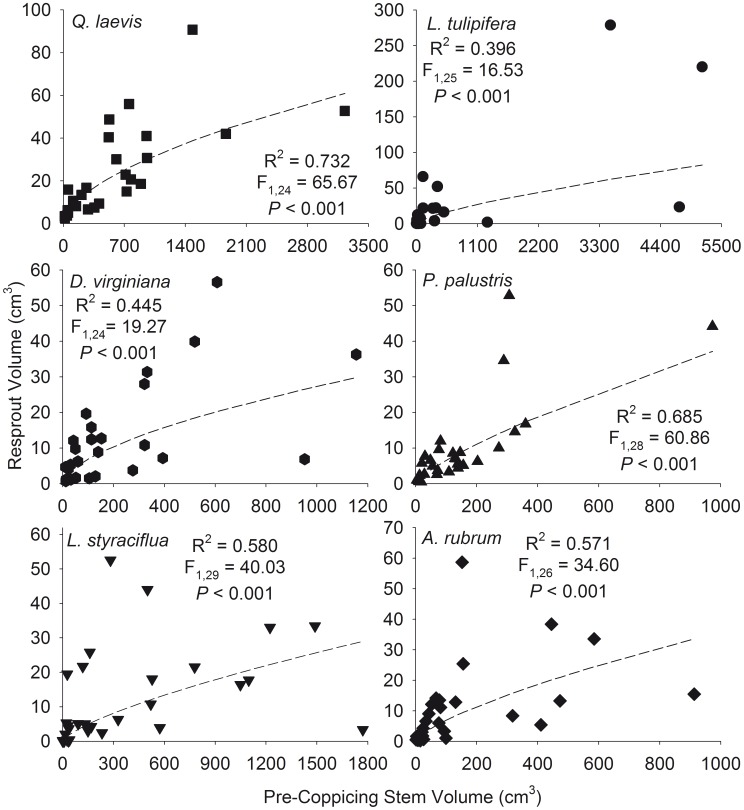
Curvilinear relationships between total pre-coppicing stem volume and total resprout stem volume.

**Figure 4:**
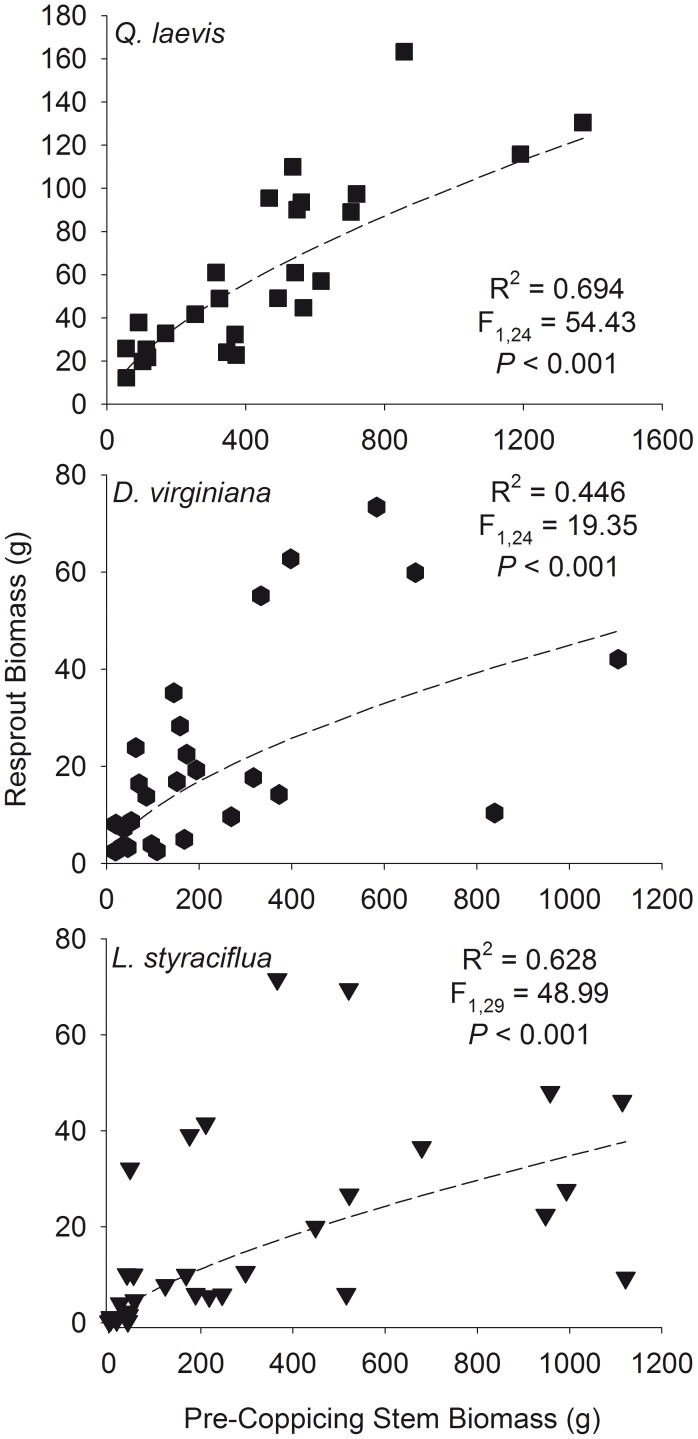
Curvilinear relationships between total pre-coppicing stem biomass and total resprout biomass.

**Figure 5:**
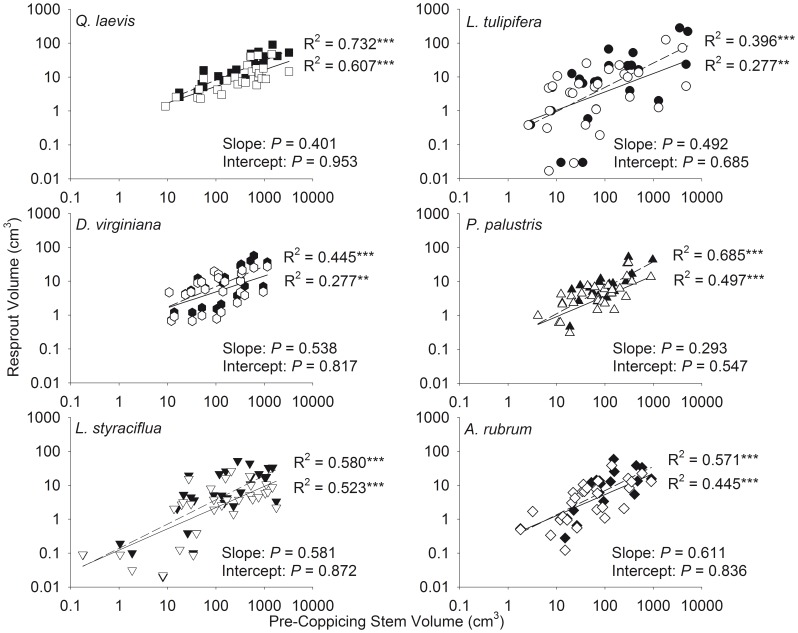
Relationships between pre-coppicing stem volume and resprout stem volume on a logarithmic scale. Total stem volume is denoted with filled symbols and dashed lines. Maximum stem volume is denoted with open symbols and solid lines. ** *P*<0.01, *** *P*<0.001 for regressions. *P* values for differences in slopes and intercepts between maximum and total volume are given.

**Figure 6:**
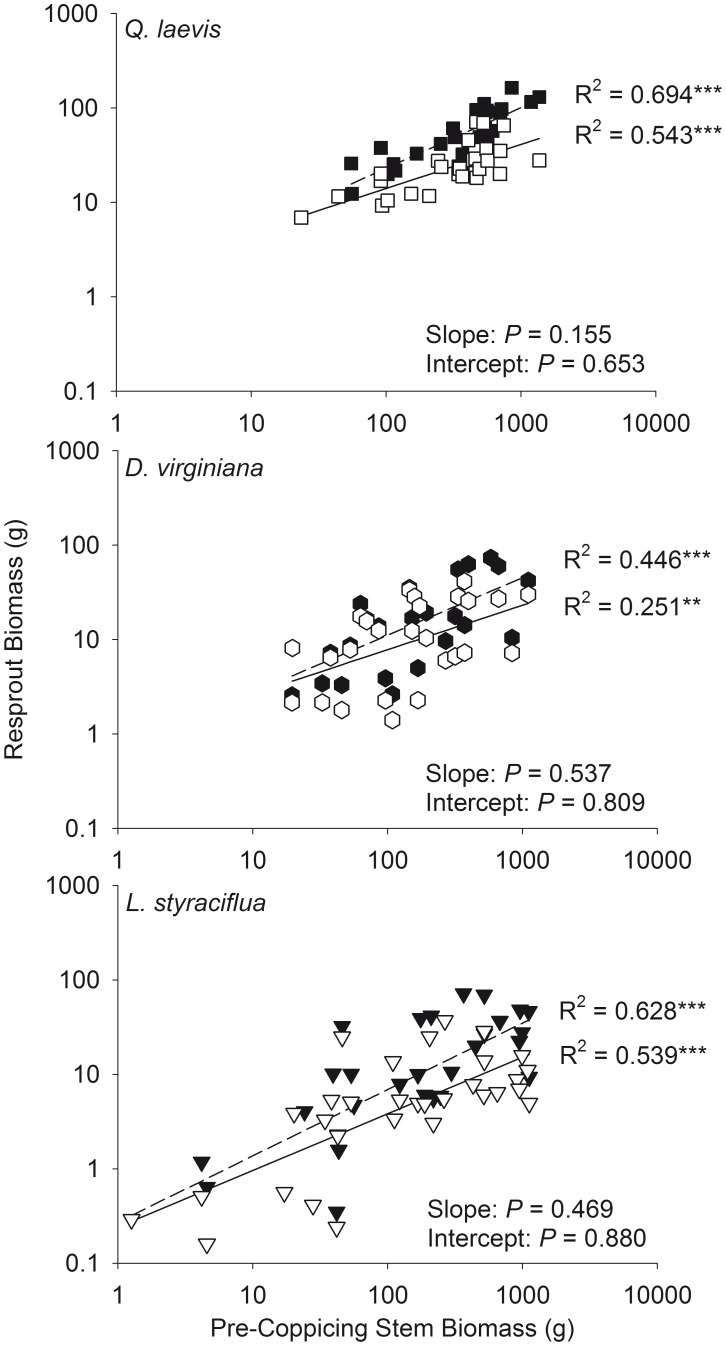
Relationships between pre-coppicing stem biomass and resprout stem biomass on a logarithmic scale. Total stem biomass is denoted with filled symbols and dashed lines. Maximum stem biomass is denoted with open symbols and solid lines. ** *P*<0.01, *** *P*<0.001 for regressions. *P* values for differences in slopes and intercepts between maximum and total biomass are given.

There was a positive curvilinear relationship between total resprout and pre-coppicing stem volume (Figure 3) and biomass (Figure 4) for all species studied. There was no difference between the slopes or intercepts of the relationships between pre-coppicing and resprout maximum and total volume (Figure 5) and pre-coppicing and resprout maximum and total biomass (Figure 6). In other words, for all species, resprout curves were similar for maximum and total size.

**Figure 7:**
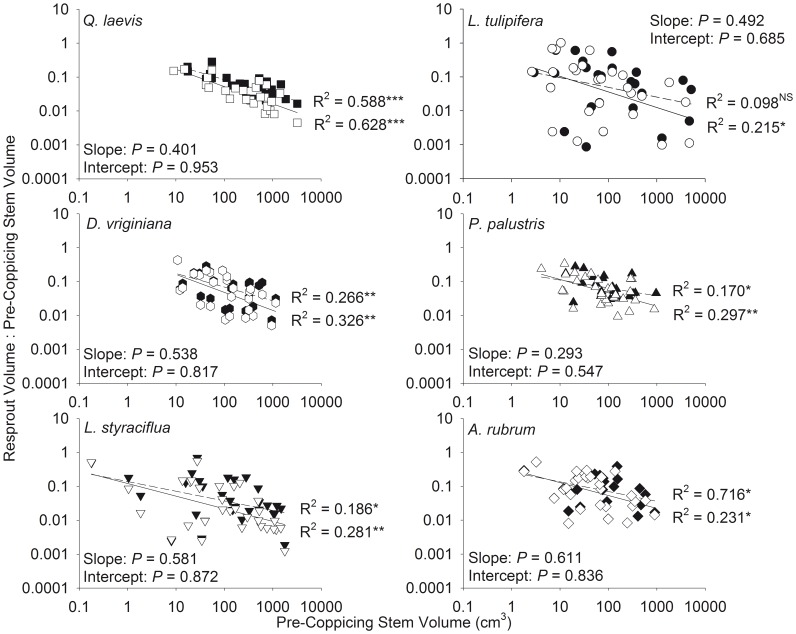
Relationships between pre-coppicing stem volume and the ratio of resprout volume to pre-coppicing volume. Data is shown on a logarithmic scale. Total stem volume is denoted with filled symbols and dashed lines. Maximum stem volume is denoted with open symbols and solid lines. * *P*<0.05, ** *P*<0.01, *** *P*<0.001 for regressions; NS indicates not significant. *P* values for differences in slopes and intercepts between maximum and total volume are given.

**Figure 8:**
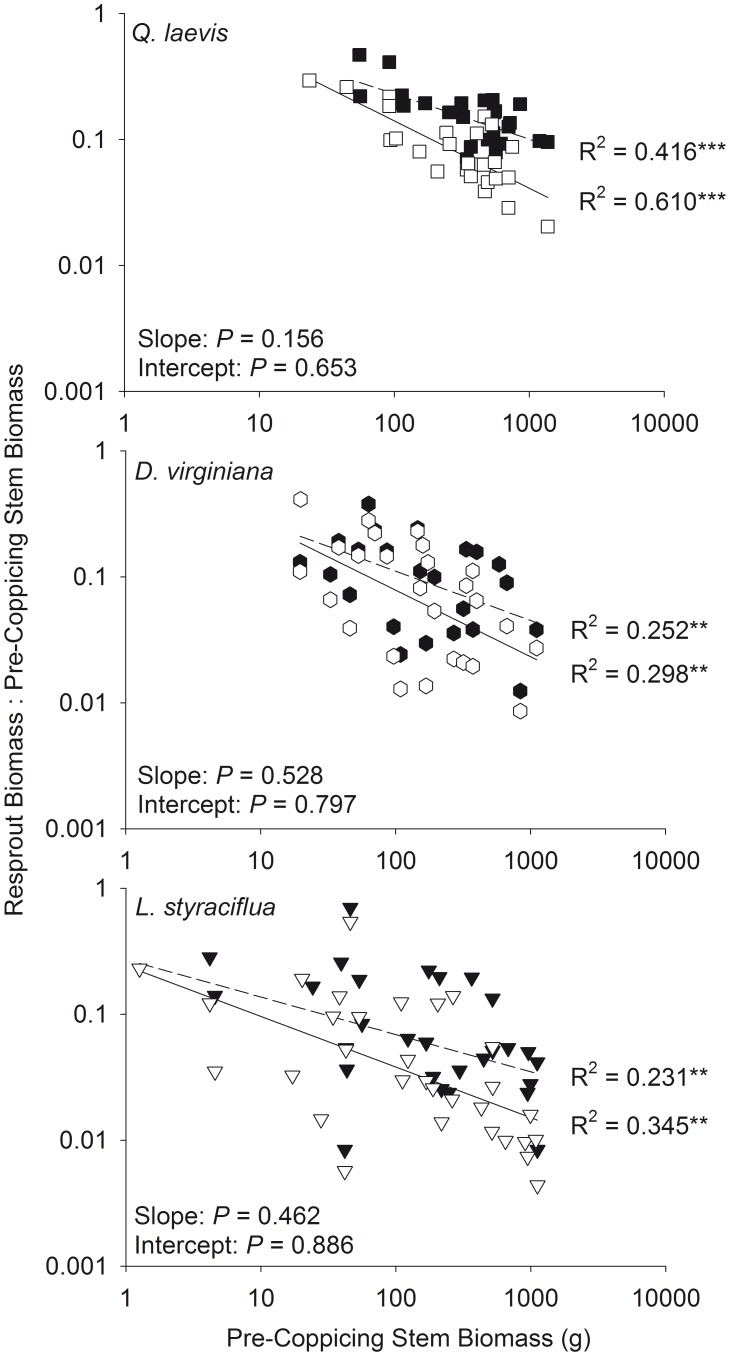
Relationships between pre-coppicing stem biomass and the ratio of resprout biomass to pre-coppicing biomass. Data is shown on a logarithmic scale. Total stem biomass is denoted with filled symbols and dashed lines. Maximum stem biomass is denoted with open symbols and solid lines. ** *P*<0.01, *** *P*<0.001 for regressions. *P* values for differences in slopes and intercepts between maximum and total biomass are given.

Recovery of volume and biomass was negatively correlated with pre-coppicing volume and biomass, regardless of whether all stems were included in the analysis. For all species, there was no difference between the slopes or intercepts of the relationships between recovery of maximum and total volume (Figure 7) and recovery of maximum and total biomass (Figure 8). Most individuals recovered less than 55% of their maximum stem volume.

## Discussion

The ability of plants to resprout after topkill contributes to their persistence in pyrogenic ecosystems. Across species in our study, 95% of individuals resprouted after complete removal of aboveground biomass (Table 1). Resprout number has been found to be positively correlated with the number of stems present before fire [20]; this was the case for four of our six study species. Individuals with more stems pre-fire may have larger storage organs, which translate to larger pools of carbohydrates and buds to support resprouting [7], [47], [48]. For the two species in our study for which resprout number was not positively correlated with pre-coppicing stem number, *D. virginiana* and *L. tulipifera*, individuals with only one stem pre-coppicing produced 1 to 5 and 1 to 14 resprouts, respectively. Factors such as bud activation and proximity of buds to the soil surface [49], as well as pre-fire stem size [15], [16], may have affected resprout number.

We found that total resprout size was positively correlated with pre-coppicing total size and fit a curvilinear function (Figures 3 and 4). Although maximum resprout size may be limited by growth rates [28], [31], the ability to increase mechanical strength to support height growth [50], or physiological changes that alter allocation of photosynthates [51], we found no difference in the resprout curves for maximum and total size (Figures 5 and 6; determined by analyzing log_10_-transformed data). Accounting for all stems did not cause a significant shift in the resprout curves, and thus, production of multiple stems does not change the size at which individuals persist in frequently burned ecosystems. One explanation for the lack of upward shift in resprout curves could be related to the concomitant increase in resprout and pre-coppicing volume and/or biomass – individuals in our study had up to eight stems pre-coppicing – such that inclusion of all stems of multi-stemmed individuals affected the location of an individual on the curve rather than the shape of the curve. In addition, intraspecific variation in pre- and post-fire sizes could be related to resource availability because plants in high-resource environments are larger after fire than plants of the same initial size in low-resource environments [18].

Similar to Grady and Hoffmann [18], when we accounted for only the largest stem, larger individuals recovered a smaller fraction of their pre-coppicing size than smaller individuals. Contrary to our hypothesis, there was no difference in the shape of the recovery curves of maximum and total size (Figures 7 and 8; determined by analyzing log_10_-transformed data). Accounting for all stems, rather than only the largest stem, does not affect which individuals, in terms of pre-coppicing size, are able to recover all biomass lost during fire. Although 73% of the resprouting individuals in our study experienced an increase in stem number after complete removal of aboveground biomass (Figure 2), our results suggest that the potential benefits of producing multiple stems do not extend to post-disturbance biomass recovery.

The shape of resprout and recovery curves may be influenced by resprout age. Historically, the fire return interval in longleaf pine savannas ranged from 0.5 to 12 years [38], and savannas at our study site are currently burned every 3 years, on average, with stream-head pocosins generally burning less frequently [39]. The number of resprouts per clump is higher in more recently burned sites than longer unburned sites [52], [53], suggesting that self-thinning of resprouts can occur over time such that total size may converge on maximum size as the number of stems decreases. Furthermore, large individuals recovered a lower proportion of their size regardless of whether resprouts were 9 months (Figures 7 and 8) or 3 years old [18].

The shape of resprout and recovery curves may also be influenced by fire season. In the southeastern US, plants burned during the dormant season have greater post-fire stem densities [4], [54] and aboveground biomass [55] than plants burned during the growing season. Differences in resprout number should have little or no effect on the persistence equilibrium since there is no difference between resprout and recovery curves of maximum and total stem size (Figures 5-8). Greater biomass [55] and a larger increase in growth rates [56] after dormant season fires suggests that resprout curves of plants burned during the dormant season could be shifted upward from plants burned during the growing season. Nonetheless, any effects of fire season on resprout and recovery curves should be consistent for maximum and total stem size.

Our study assessed the importance of considering all stems, not just the largest stems, when assessing size recovery after complete removal of aboveground biomass. The ability of plants to produce multiple resprouts did not allow individuals to reach a larger equilibrium size or recover a greater proportion of their pre-coppicing size. Production of multiple stems, therefore, does not appear to affect persistence in frequently burned ecosystems in regard to the balance between biomass loss and recovery. Our study species are all trees that resprouted from the root crown; it is not clear how differences in allometric constraints on resprout allocation (e.g., shrubs vs. trees; [57]) or the belowground structure from which resprouts are produced (e.g., lignotubers or rhizomes; [3]) influence relationships between maximum and total stem size. Nevertheless, accounting for only the largest pre-fire stem and largest resprout appears to be an adequate predictor of species' equilibrium size and their ability to recovery their pre-fire size and should not lead to misinterpretation of persistence ability over multiple fire cycles.

## Supporting Information

Dataset S1 **Pre-coppicing and resprout stem volume and biomass.**
(XLSX)
